# Preliminary Phytochemical Screening and Antimicrobial Studies of *Lantana indica* Roxb

**DOI:** 10.4103/0250-474X.65020

**Published:** 2010

**Authors:** R. Venkataswamy, A. Doss, M. Sukumar, H. M. Mubarack

**Affiliations:** Department of Pharmacognosy, SRIPMS College of Pharmacy, Coimbatore-641 044, India; 1 Department of Microbiology, RVS College of Arts and Science, Sulur, Coimbatore-641 402, India

**Keywords:** *Lantana indica*, agar well diffusion method, antimicrobial activity

## Abstract

The aim of the present study was to investigate the antimicrobial and preliminary phytochemical properties of *Lantana indica* Roxb. The aqueous and organic solvent (ethyl acetate and methanol) extracts from the leaves of *Lantana indica* (Verbenaceae) were tested against *Staphylococcus aureus, Bacillus subtilis, Steptococcus pyrogens, Escherichia coli, Proteus vulgaris, Klebsiella pneumoniae, Pseudomonas aeruginosa, Salmonella typhi, Aspergillus niger* and *Candida albicans* by agar well diffusion method. The results showed promising antibacterial activity against the tested bacteria. Among these, methanol and aqueous extracts were found to possess a more potent inhibitory effect when compared to the ethyl acetate extract. Preliminary phytochemical analysis of extracts revealed the presence of antimicrobial compounds such as carbohydrates, proteins, tannins and flavonoidal glycosides. The result of this study validates the use of methanol and aqueous extract of this species in ethnomedicine, favouring the isolation of antibacterial agents from the leaf extract of *Lantana indica*.

Medicinal plants are an important source for the therapeutic remedies of various ailments. Scientific experiments on the antimicrobial properties of plant components were first documented in the late 19^th^ century[1]. Since time immemorial, different parts of medicinal plants have been used to cure specific ailments in India. Now-a-days there is widespread interest in evaluating drugs derived from plant sources. This interest primarily stems from the belief that green medicine is safe and dependable, compared to costly synthetic drugs which are invariably associated with adverse effects. Natural antimicrobials have been often derived from plants, animal tissues or microorganisms[2]. The adverse effects of the drugs available today, necessitates the discovery of new harmless pharmacotherapeutic agents from medicinal plants[3].

*Lantana*, belonging to family Verbenaceae, is a genus of about 150 species, mostly native to subtropical and tropical America. But a few taxa are indigenous to tropical Asia and Africa. The genus is a complex one to classify taxonomically, since the species are not stable due to widespread hybridization[4–6]. *Lantana indica* Roxb. is a wild shrub, native to India, with bunches of light purple flowers with opposite and whorled pubescent leaves. It is regarded both as a notorious weed and a popular ornamental garden plant. It is used in Indian medicine as a sudorific, intestinal antiseptic and diaphoretic besides its employment in the treatment of tetanus, rheumatism and malaria[6–8]. Phytochemically, the plant has revealed the presence of several triterpenes[8–11] and sterols[11]. The present study was aimed at the preliminary phytochemical screening and exploration of antimicrobial properties in the aqueous and organic solvent extracts of the leaves of *Lantana indica*.

Leaves of *L. indica* were collected from Ayyasamy hills of Tamilnadu in the month of November 2008. The plant was identified and authenticated by Botanical Survey of India (Southern Circle), Coimbatore, Tamilnadu and a voucher specimen was deposited in the Herbarium of Department of Botany, Government Arts College, Coimbatore, Tamilnadu. The collected plant materials were shade dried at room temperature and mechanically reduced to coarse powder form. The leaf powder (250 g) was then extracted individually with methanol in a Soxhlet apparatus by continuous hot extraction for 72 h. Simultaneously, 250 g of dried leaf powder was placed in 500 ml of water and macerated for 24 h. The extracts so obtained were concentrated to dryness by evaporating the solvents under reduced pressure.

The antibacterial and antifungal studies of the aqueous and organic solvent extracts of the leaves were carried out by the agar well diffusion method[12]. The extracts were individually dissolved in dimethylsulfoxide (DMSO) to get 10 mg/ml solution. Ciprofloxacin (1 mg/ml) and fluconazole (1 mg/ml) were used as standard antibacterial and antifungal agents separately. The antibacterial activity was evaluated by employing 24 h cultures of *Bacillus subtilis, Staphylococcus aureus, Streptococcus pyrogens, E. coli, Proteus vulgaris, Klebsiella pneumonia, Salmonella typhi* and *Pseudomonas aeruginosa* using Muller Hinton Agar medium. Antifungal activity was carried out against 24 h cultures of *Candida albicans* and *Aspergillus niger* using Sabouraud Dextrose Agar medium. The bacterial and fungal strains employed in the study were obtained from NCIM, Pune. 0.2 ml of the test and standard solutions were transferred to wells in the microorganisms inoculated plates aseptically and labeled accordingly. Then the plates were maintained at room temperature for 2 h enabling the diffusion of the solutions into the medium. The petriplates used for antibacterial screening were incubated at 37±1° for 24 h, while those used for antifungal activity were incubated at 28±1° for 48 h. The diameters of zone of inhibition surrounding each of the wells were recorded. The preliminary phytochemical studies were carried out by the methods described by Harborne and Kokate *et al*. The plant extracts were screened for the presence of proteins, triterpenoids, carbohydrates, flavonoidal glycosides and tannins.

Tables 1 and 2 show the antibacterial and antifungal activity of the extracts of *L. indica*. The methanol and aqueous extracts of the leaves clearly exhibited strong activity against the tested microorganisms, while the ethyl acetate extract showed moderate activity. The results revealed that, the methanol and aqueous extracts of leaves exhibited strong inhibitory action against *Bacillus subtilis, Staphylococcus aureus, Streptococcus pyrogens, E. coli, Proteus vulgaris, Klebsiella pneumoniae* and *Candida albicans* and moderate inhibitory action against *Pseudomonas aeruginosa, Salmonella typhi* and *Aspergillus niger*. The results of preliminary phytochemical screening of methanol, ethyl acetate and aqueous leaf extracts of *Lantana indica* are presented inTable 3. The methanol and aqueous extracts showed the presence of flavonoidal glycosides, carbohydrates, proteins, triterpenoids and tannins.

**TABLE 1:** ANTIBACTERIAL SCREENING OF *LANTANA INDICA* ROXB

Microorganisms	Zone of inhibition (mm)									Ciprofloxacin (25 µg)
	Methanol (µg)			Ethyl acetate (µg)			Aqueous (µg)		
	2000	1500	1000	2000	1500	1000	2000	1500	1000
*Bacillus subtilis*	21	15	11	14	10	-	21	14	-	28
*Staphylococcus aureus*	21	12	-	15	11	9	21	12	-	29
*Streptococcus pyrogens*	18	11	-	12	9	-	19	11		24
*E. coli*	21	17	12	11	8	-	20	13	-	30
*Proteus vulgaris*	19	13	10	10	9	-	20	12	-	22
*Pseudomonas aeruginosa*	13	9	-	12	9	-	10	8	-	27
*Salmonella typhi*	12	9	-	10	8	-	12	8		21
*Klebsiella pneumonia*	15	11	10	14	10	-	18	11	10	20

**TABLE 2:** ANTIFUNGAL SCREENING OF *LANTANA INDICA* ROXB

Microorganisms	Zone of inhibition (in mm)									Fluconazole (25 µg)
	Methanol (µg)			Ethyl acetate (µg)			Aqueous (µg)		
	2000	1500	1000	2000	1500	1000	2000	1500	1000
*Aspergillus niger*	14	-	-	10	-	-	-	-	-	29
*Candida albicans*	12	-	-	9	-	-	-	-	-	28

**TABLE 3:** PRELIMINARY PHYTOCHEMICAL SCREENING OF *LANTANA INDICA* ROXB

Extracts	Carbohydrates	Proteins	Glycosidess	Alkaloids	Flavonoids	Tannins	Triterpenoids
Methanol	+	+	+	−	+	+	+
Ethyl acetate	+	−	+	−	+	−	+
Aqueous	+	+	+	−	+	+	+

Several plants, rich in tannins have been shown to possess antimicrobial activities against a number of microorganisms. For example Banso and Adeyemo[13] investigated the antibacterial activity of leaf extract of *Dichrostachys cinerea* and reported the presence of tannins, alkaloids and glycosides. Amongst the gram-positive and gram-negative bacteria, gram-positive bacterial strains were more susceptible to the extracts when compared to gram negative bacteria. This may be attributed to the fact that these two groups differ in their structure of the cell wall components[14]. The ability of tannin compounds to cause the bacterial colonies to disintegrate, probably results from their interference with the bacterial cell wall; thereby inhibiting the microbial growth[15,16]. The results of the present study support the traditional use of the *Lantana indica* as an ethnomedicine. It also suggests that the tannins isolated from the *Lantana indica* possess remarkable antimicrobial activity against microbial pathogens.
